# Supplementary material for: Viral respiratory infections and the oropharyngeal bacterial microbiota in acutely wheezing children
Source: PLoS One. 2019 Oct 17;14(10):e0223990. doi: 10.1371/journal.pone.0223990 (PMC6797130; doi:10.1371/journal.pone.0223990)
Supplement: S1 Table — Data is recorded as n (%) or median (min-max). Data is recorded as a percentage of all data collected. (DOCX) [file pone.0223990.s001.docx]

S1 Table. Participant demographics continued. Data is recorded as n (%) or median (min-max). Data is recorded as a percentage of all data collected.

|  | **Acute** | | **Controls** | |
| --- | --- | --- | --- | --- |
| **Ethnic group** | **N** | **All** | **N** | **All** |
| Aboriginal (%) | 109 | 5 (5%) | 70 | 2 (35%) |
| African/African American (%) |  | 12 (11%) |  | 1 (1%) |
| Asian/Indian (%) |  | 23 (21%) |  | 16 (21%) |
| Caucasian (%) |  | 54 (50%) |  | 51 (68%) |
| Maori (%) |  | 3 (3%) |  | 0 |
| Pacific Islanders/Samoan (%) |  | 2 (2%) |  | 0 |
| PNG (%) |  | 2 (2%) |  | 0 |
| Undetermined (%) |  | 8 (7%) |  | 5 (7%) |
| **Season** |  |  |  |  |
| Autumn (%) | 109 | 20 (18%) | 75 | 4 (5%) |
| Spring (%) |  | 18 (16%) |  | 17 (23%) |
| Summer (%) |  | 3 (3%) |  | 3 (4%) |
| Winter (%) |  | 68 (62%) |  | 51 (68%) |
| **Medication** |  |  |  |  |
| Systemic steroids (%) | 105 | 76 (72%) | 67 | 0 |
| Oxygen (%) | 99 | 45 (45%) |  |  |
| **Blood counts** |  |  |  |  |
| Platelets (min-max) | 82 | 295 (105 - 611) | 41 | 292 (35 - 655) |
| T-cells (min-max) | 83 | 10.5 (3.5 - 27.9) | 41 | 8.3 (4 - 13.4) |
| Monocytes (min-max) | 83 | 0.54 (0.275 - 3.39) | 40 | 0.685 (0.3 - 2.18) |
| Eosinophils (min-max) | 83 | 0.06 (0.015 - 2.67) | 40 | 0.28 (0 - 1.42) |
| Basophils (min-max) | 83 | 0.01 (0 - 0.38) | 40 | 0.05 (0 - 0.18) |
| **Severity** |  |  |  |  |
| Severity Z-score (min-max) | 85 | 0.3191 (-2.2306 - 2.0151) |  |  |
| O2 Saturation | 89 | 95 (69 - 99) |  |  |
|  |  |  |  |  |
