# Supplementary material for: Viral respiratory infections and the oropharyngeal bacterial microbiota in acutely wheezing children
Source: PLoS One. 2019 Oct 17;14(10):e0223990. doi: 10.1371/journal.pone.0223990 (PMC6797130; doi:10.1371/journal.pone.0223990)
Supplement: S2 Table — P values adjusted using Bonferonni correction for multiple testing. (DOCX) [file pone.0223990.s002.docx]

S2 Table. Comparison of categorical clinical variables with Wilcoxon rank sum test in the complete cohort to alpha diversity measures, richness, Shannon-Weiner and Simpsons reciprocal. P values adjusted using Bonferonni correction for multiple testing.

|  | Richness | | | Shannon-Weiner | | | Inverse Simpsons | | |
| --- | --- | --- | --- | --- | --- | --- | --- | --- | --- |
| Clinical variable | W | p | p-adjusted | W | p | p-adjusted | W | p | p-adjusted |
| Case (Wheeze) | 4278.5 | 0.591 | 1 | 4081 | 0.987 | 1 | 3933 | 0.664 | 1 |
| Gender | 4152.5 | 0.933 | 1 | 4408 | 0.531 | 1 | 4421 | 0.507 | 1 |
| Asthmas exacerbation | 1469 | 0.077 | 1 | 1620 | 0.302 | 1 | 1724 | 0.601 | 1 |
| Wheezy episode | 421.5 | 0.742 | 1 | 299 | 0.338 | 1 | 288 | 0.282 | 1 |
| Viral wheeze | 2044 | 0.693 | 1 | 1941 | 0.389 | 1 | 1860 | 0.219 | 1 |
| Pneumonia | 20 | 0.231 | 1 | 17 | 0.203 | 1 | 25 | 0.285 | 1 |
| Bronchiolitis | 1795 | 0.000 | 0.006 | 1744 | 0.001 | 0.017 | 1678 | 0.002 | 0.062 |
| URTI | 3389.5 | 0.293 | 1 | 3325 | 0.403 | 1 | 3322 | 0.409 | 1 |
| Oxygen required | 1642.5 | 0.003 | 0.089 | 1694 | 0.001 | 0.025 | 1719 | 0.000 | 0.013 |
| Systemic steroids | 3269 | 0.243 | 1 | 3224 | 0.192 | 1 | 3191 | 0.159 | 1 |
| RV | 3693 | 0.193 | 1 | 4035 | 0.728 | 1 | 4159 | 0.999 | 1 |
| RSV | 1560 | 0.003 | 0.104 | 1499 | 0.010 | 0.337 | 1364 | 0.085 | 1 |
| Adenovirus | 764.5 | 0.125 | 1 | 689 | 0.376 | 1 | 641 | 0.635 | 1 |
| Influenza virus | 91 | 0.592 | 1 | 110 | 0.313 | 1 | 110 | 0.313 | 1 |
| Parainfluenza virus | 152.5 | 0.460 | 1 | 170 | 0.627 | 1 | 175 | 0.680 | 1 |
| Mycoplasma | 370 | 0.031 | 1 | 313 | 0.194 | 1 | 319 | 0.165 | 1 |
| Bordatella | 458 | 0.954 | 1 | 483 | 0.750 | 1 | 472 | 0.839 | 1 |
| Corona Virus | 98 | 0.322 | 1 | 47 | 0.045 | 1 | 80 | 0.178 | 1 |
| hMPV | 369 | 0.202 | 1 | 390 | 0.123 | 1 | 369 | 0.202 | 1 |
| Enterovirus | 118.5 | 0.806 | 1 | 120 | 0.771 | 1 | 124 | 0.669 | 1 |
| Bocavirus | 185.5 | 0.888 | 1 | 207 | 0.739 | 1 | 189 | 0.954 | 1 |
| Pathogen positive | 3319.5 | 0.683 | 1 | 3519 | 0.294 | 1 | 3585 | 0.207 | 1 |
| Virus positive | 3266 | 0.978 | 1 | 3489 | 0.498 | 1 | 3555 | 0.375 | 1 |
| nonRV positive | 2710 | 0.011 | 0.351 | 2611 | 0.035 | 1 | 2485 | 0.122 | 1 |
| Atopy | 2489 | 0.142 | 1 | 2457 | 0.113 | 1 | 2431 | 0.093 | 1 |
| Smoking now | 2070 | 0.049 | 1 | 2095 | 0.038 | 1 | 2114 | 0.031 | 1 |
| Smoking when pregnant | 1973 | 0.195 | 1 | 2081 | 0.076 | 1 | 2041 | 0.110 | 1 |
| Smoking regularly when pregnant | 1393.5 | 0.715 | 1 | 1447 | 0.527 | 1 | 1400 | 0.691 | 1 |
| Household smoking | 3506 | 0.121 | 1 | 3434 | 0.189 | 1 | 3370 | 0.269 | 1 |
| Kindergarten | 2846 | 0.002 | 0.082 | 2728 | 0.001 | 0.025 | 2699 | 0.001 | 0.018 |
| Pre-school | 2437 | 0.006 | 0.186 | 2507 | 0.011 | 0.359 | 2561 | 0.017 | 0.577 |
| Daycare | 3329.5 | 0.023 | 0.766 | 3263 | 0.014 | 0.463 | 3455 | 0.055 | 1 |
