# Supplementary material for: Viral respiratory infections and the oropharyngeal bacterial microbiota in acutely wheezing children
Source: PLoS One. 2019 Oct 17;14(10):e0223990. doi: 10.1371/journal.pone.0223990 (PMC6797130; doi:10.1371/journal.pone.0223990)
Supplement: S3 Table — P values adjusted using Bonferonni correction for multiple testing. (DOCX) [file pone.0223990.s003.docx]

S3 Table. Pearson’s correlations of continuous clinical variables and alpha diversity measures, richness, Shannon-Weiner and Simpsons reciprocal, in the complete cohort. P values adjusted using Bonferonni correction for multiple testing.

|  | Richness | | | Shannon-Weiner | | | Inverse Simpsons | | |
| --- | --- | --- | --- | --- | --- | --- | --- | --- | --- |
| Clinical variable | r | p | p-adjusted | r | p | p-adjusted | r | p | p-adjusted |
| Bacterial Biomass | 0.2438 | 0.0009 | 0.0210 | 0.169 | 0.023 | 0.521 | 0.161 | 0.030 | 0.694 |
| Age | 0.1840 | 0.0126 | 0.2909 | 0.168 | 0.023 | 0.525 | 0.147 | 0.046 | 1 |
| Ethnic group | 0.0021 | 0.9777 | 1 | -0.049 | 0.511 | 1 | -0.059 | 0.434 | 1 |
| Platelets | -0.0264 | 0.7721 | 1 | 0.014 | 0.881 | 1 | 0.017 | 0.855 | 1 |
| T-cell count | 0.0075 | 0.9341 | 1 | 0.094 | 0.298 | 1 | 0.077 | 0.396 | 1 |
| Neutraphils | 0.0933 | 0.3045 | 1 | 0.211 | 0.019 | 0.439 | 0.184 | 0.042 | 0.965 |
| Lymphocytes | -0.1783 | 0.0485 | 1 | -0.250 | 0.005 | 0.122 | -0.228 | 0.011 | 0.262 |
| Monocytes | -0.1935 | 0.0320 | 0.737 | -0.177 | 0.051 | 1 | -0.151 | 0.096 | 1 |
| Eosinophils | 0.2359 | 0.0086 | 0.198 | 0.218 | 0.015 | 0.350 | 0.157 | 0.083 | 1 |
| Basophils | 0.1825 | 0.0434 | 0.998 | 0.110 | 0.227 | 1 | 0.088 | 0.333 | 1 |
| Time to 1 hourly Ventolin | -0.0960 | 0.3940 | 1 | -0.103 | 0.360 | 1 | -0.100 | 0.373 | 1 |
| Severity Z-score | -0.0765 | 0.4864 | 1 | -0.137 | 0.210 | 1 | -0.150 | 0.171 | 1 |
| O2 Saturation | 0.1206 | 0.2604 | 1 | 0.190 | 0.075 | 1 | 0.175 | 0.102 | 1 |
| Total IgE | -0.2378 | 0.1036 | 1 | -0.140 | 0.344 | 1 | -0.024 | 0.872 | 1 |
| House dust mite IgE | 0.0587 | 0.6917 | 1 | 0.154 | 0.295 | 1 | 0.167 | 0.256 | 1 |
| Cat IgE | -0.0684 | 0.6440 | 1 | -0.168 | 0.254 | 1 | -0.162 | 0.271 | 1 |
| Cathlecidin | 0.0517 | 0.6951 | 1 | 0.100 | 0.446 | 1 | 0.119 | 0.363 | 1 |
| Gestation period | 0.0182 | 0.8141 | 1 | 0.021 | 0.790 | 1 | -0.003 | 0.973 | 1 |
| No of children | 0.0096 | 0.8979 | 1 | -0.049 | 0.515 | 1 | -0.069 | 0.357 | 1 |
| No of siblings | -0.0937 | 0.2108 | 1 | -0.095 | 0.206 | 1 | -0.094 | 0.208 | 1 |
