# Supplementary material for: Viral respiratory infections and the oropharyngeal bacterial microbiota in acutely wheezing children
Source: PLoS One. 2019 Oct 17;14(10):e0223990. doi: 10.1371/journal.pone.0223990 (PMC6797130; doi:10.1371/journal.pone.0223990)
Supplement: S4 Table — P values adjusted using Bonferonni correction for multiple testing. (DOCX) [file pone.0223990.s004.docx]

S4 Table. Results of Bray-Curtis adonis permutational ANOVA examining clinical variables with 99,999 iterations. P values adjusted using Bonferonni correction for multiple testing.

| **Clinical variable** | **R^2^** | **P** | **p-adjusted** |
| --- | --- | --- | --- |
| Bacterial biomass | 0.06 | 0 | 0 |
| Case (Wheeze) | 0.016 | 0.007 | 0.434 |
| Asthma exacerbation | 0.011 | 0.155 | 1 |
| Wheezy episode | 0.009 | 0.257 | 1 |
| viral wheeze | 0.01 | 0.214 | 1 |
| Bronchiolitis | 0.034 | 0 | 0 |
| URTI | 0.01 | 0.088 | 1 |
| Age | 0.014 | 0.018 | 1 |
| Season | 0.028 | 0.024 | 1 |
| Systemic steroids | 0.012 | 0.051 | 1 |
| Ethnic group | 0.004 | 0.66 | 1 |
| Platelets | 0.006 | 0.684 | 1 |
| T-cell count | 0.016 | 0.053 | 1 |
| Neutraphils | 0.028 | 0.004 | 0.248 |
| Lymphocytes | 0.016 | 0.056 | 1 |
| Monocytes | 0.015 | 0.068 | 1 |
| Eosinophils | 0.009 | 0.287 | 1 |
| Basophils | 0.004 | 0.902 | 1 |
| Oxygen required | 0.039 | 0.002 | 0.124 |
| Severity Z-score | 0.018 | 0.142 | 1 |
| O2saturation | 0.012 | 0.361 | 1 |
| RV | 0.003 | 0.777 | 1 |
| RV strain | 0.004 | 0.689 | 1 |
| RSV | 0.011 | 0.118 | 1 |
| Adenovirus | 0.006 | 0.551 | 1 |
| Influenza virus | 0.005 | 0.733 | 1 |
| Parainfluenza | 0.004 | 0.871 | 1 |
| Mycoplasma | 0.014 | 0.115 | 1 |
| Bordatella | 0.01 | 0.296 | 1 |
| Corona Virus | 0.005 | 0.904 | 1 |
| hMPV | 0.009 | 0.222 | 1 |
|  |  |  |  |
| **Clinical variable** | **R^2^** | **P** | **p-adjusted** |
| Enterovirus | 0.038 | 0.101 | 1 |
| No positive pathogens | 0.006 | 0.386 | 1 |
| Pathogens tested | 0.009 | 0.104 | 1 |
| Pathogen positive | 0.004 | 0.711 | 1 |
| No viruses tested | 0.011 | 0.057 | 1 |
| No virus positive | 0.003 | 0.79 | 1 |
| Positive for virus not RV | 0.009 | 0.222 | 1 |
| No positive viruses, not RV | 0.006 | 0.631 | 1 |
| No positive viruses | 0.004 | 0.592 | 1 |
| Atopy | 0.015 | 0.024 | 1 |
| Total IgE | 0.012 | 0.847 | 1 |
| House dust mite IgE | 0.02 | 0.477 | 1 |
| Cat IgE | 0.024 | 0.397 | 1 |
| Antibiotics | 0.009 | 0.157 | 1 |
| Cathlecidin | 0.018 | 0.356 | 1 |
| Current Smoking now | 0.01 | 0.069 | 1 |
| Smoking when pregnant | 0.011 | 0.048 | 1 |
| Smoking regularly when pregnant | 0.005 | 0.481 | 1 |
| Household smoking | 0.007 | 0.238 | 1 |
| Gestation period | 0.008 | 0.211 | 1 |
| No children | 0.005 | 0.473 | 1 |
| No siblings | 0.009 | 0.129 | 1 |
| Kindergarten | 0.031 | 0 | 0 |
| Preschool | 0.022 | 0.001 | 0.062 |
| Daycare | 0.007 | 0.189 | 1 |
| Recurrence | 0.045 | 0.052 | 1 |
|  |  |  |  |
