# Supplementary material for: Viral respiratory infections and the oropharyngeal bacterial microbiota in acutely wheezing children
Source: PLoS One. 2019 Oct 17;14(10):e0223990. doi: 10.1371/journal.pone.0223990 (PMC6797130; doi:10.1371/journal.pone.0223990)
Supplement: S5 Table — P values adjusted using Bonferonni correction for multiple testing. (DOCX) [file pone.0223990.s005.docx]

S5 Table. Comparison of categorical clinical variables in those with acute wheeze to alpha diversity measures, richness, Shannon-Weiner and inverse Simpsons. P values adjusted using Bonferonni correction for multiple testing.

|  | Richness | | | Shannon-Weiner | | | Inverse Simpsons | | |
| --- | --- | --- | --- | --- | --- | --- | --- | --- | --- |
| Clinical variable | W | p | p-adjusted | W | p | p-adjusted | W | p | p-adjusted |
| Gender | 1524 | 0.787 | 1 | 1604 | 0.450 | 1 | 1635 | 0.345 | 1 |
| Asthmas exacerbation | 985 | 0.027 | 0.796 | 1163 | 0.281 | 1 | 1273 | 0.708 | 1 |
| Wheezy episode | 306 | 0.973 | 1 | 230 | 0.297 | 1 | 236 | 0.335 | 1 |
| Viral wheeze | 1439.5 | 0.855 | 1 | 1335 | 0.413 | 1 | 1289 | 0.272 | 1 |
| Pneumonia | 29.5 | 0.445 | 1 | 22 | 0.317 | 1 | 29 | 0.436 | 1 |
| Bronchiolitis | 1056 | 0.000 | 0.002 | 1021 | 0.000 | 0.006 | 984 | 0.001 | 0.023 |
| URTI | 775.5 | 0.538 | 1 | 725 | 0.864 | 1 | 773 | 0.553 | 1 |
| Oxygen required | 1620.5 | 0.004 | 0.132 | 1712 | 0.000 | 0.015 | 1730 | 0.000 | 0.009 |
| Systemic steroids | 653.5 | 0.001 | 0.040 | 763 | 0.015 | 0.458 | 819 | 0.043 | 1 |
| RV | 1384 | 0.826 | 1 | 1449 | 0.527 | 1 | 1476 | 0.421 | 1 |
| RSV | 696.5 | 0.006 | 0.177 | 668 | 0.017 | 0.507 | 596 | 0.141 | 1 |
| Adenovirus | 162 | 0.165 | 1 | 116 | 0.873 | 1 | 104 | 0.894 | 1 |
| Parainfluenza virus | 20 | 0.438 | 1 | 18 | 0.386 | 1 | 19 | 0.412 | 1 |
| Mycoplasma | 89 | 0.166 | 1 | 80 | 0.317 | 1 | 86 | 0.209 | 1 |
| Bordatella | 28 | 0.241 | 1 | 27 | 0.225 | 1 | 19 | 0.120 | 1 |
| Corona Virus | 29 | 0.633 | 1 | 6 | 0.043 | 1 | 15 | 0.194 | 1 |
| hMPV | 170 | 0.517 | 1 | 201 | 0.168 | 1 | 198 | 0.191 | 1 |
| Enterovirus | 54 | 0.670 | 1 | 54 | 0.688 | 1 | 54 | 0.688 | 1 |
| Bocavirus | 0 | 0.200 | 1 | 0 | 0.200 | 1 | 0 | 0.200 | 1 |
| Pathogen positive | 1144 | 0.047 | 1 | 1136 | 0.055 | 1 | 1152 | 0.041 | 1 |
| Virus positive | 1138.5 | 0.100 | 1 | 1125 | 0.123 | 1 | 1139 | 0.099 | 1 |
| nonRV positive | 909.5 | 0.011 | 0.320 | 830 | 0.090 | 1 | 764 | 0.327 | 1 |
| Atopy | 733 | 0.076 | 1 | 748 | 0.099 | 1 | 763 | 0.127 | 1 |
| Smoking now | 1031.5 | 0.068 | 1 | 1043 | 0.055 | 1 | 1066 | 0.035 | 1 |
| Smoking when pregnant | 901.5 | 0.359 | 1 | 927 | 0.258 | 1 | 924 | 0.269 | 1 |
| Smoking regularly when pregnant | 737 | 0.624 | 1 | 709 | 0.814 | 1 | 693 | 0.928 | 1 |
| Household smoking | 1451 | 0.195 | 1 | 1475 | 0.146 | 1 | 1459 | 0.177 | 1 |
| Kindergarten | 773 | 0.000 | 0.001 | 820 | 0.000 | 0.004 | 864 | 0.000 | 0.013 |
| Pre-school | 809 | 0.000 | 0.012 | 925 | 0.005 | 0.151 | 1026 | 0.031 | 0.924 |
| Daycare | 1308 | 0.585 | 1 | 1204 | 0.229 | 1 | 1225 | 0.285 | 1 |
