# Supplementary material for: Viral respiratory infections and the oropharyngeal bacterial microbiota in acutely wheezing children
Source: PLoS One. 2019 Oct 17;14(10):e0223990. doi: 10.1371/journal.pone.0223990 (PMC6797130; doi:10.1371/journal.pone.0223990)
Supplement: S6 Table — richness, Shannon-Weiner and inverse Simpsons. P values adjusted using Bonferonni correction for multiple testing. (DOCX) [file pone.0223990.s006.docx]

S6 Table. Pearson’s correlations of continuous clinical variables in those with acute wheeze and alpha diversity measures. richness, Shannon-Weiner and inverse Simpsons reciprocal. P values adjusted using Bonferonni correction for multiple testing

|  | Richness | | | Shannon-Weiner | | | Inverse Simpsons | | |
| --- | --- | --- | --- | --- | --- | --- | --- | --- | --- |
| Clinical variable | r | p | p-adjusted | r | p | p-adjusted | r | p | p-adjusted |
| Bacterial biomass | 0.290 | 0.002 | 0.056 | 0.207 | 0.032 | 0.737 | 0.202 | 0.037 | 0.845 |
| Age | 0.375 | 0.000 | 0.001 | 0.283 | 0.003 | 0.067 | 0.220 | 0.021 | 0.489 |
| Ethnic group | 0.052 | 0.593 | 1 | -0.041 | 0.673 | 1 | -0.059 | 0.541 | 1 |
| Platelets | -0.049 | 0.662 | 1 | -0.036 | 0.750 | 1 | 0.002 | 0.985 | 1 |
| T-cell count | 0.064 | 0.565 | 1 | 0.122 | 0.273 | 1 | 0.097 | 0.383 | 1 |
| Neutraphils | 0.174 | 0.115 | 1 | 0.244 | 0.026 | 0.608 | 0.218 | 0.048 | 1 |
| Lymphocytes | -0.260 | 0.018 | 0.406 | -0.301 | 0.006 | 0.133 | -0.282 | 0.010 | 0.225 |
| Monocytes | -0.276 | 0.012 | 0.268 | -0.210 | 0.057 | 1 | -0.173 | 0.117 | 1 |
| Eosinophils | 0.310 | 0.004 | 0.100 | 0.289 | 0.008 | 0.187 | 0.188 | 0.088 | 1 |
| Basophils | 0.263 | 0.016 | 0.376 | 0.131 | 0.239 | 1 | 0.087 | 0.433 | 1 |
| Time to 1 hourly Ventolin | -0.069 | 0.538 | 1 | -0.093 | 0.407 | 1 | -0.101 | 0.368 | 1 |
| Severity Z-score | -0.037 | 0.737 | 1 | -0.118 | 0.280 | 1 | -0.140 | 0.201 | 1 |
| O2 Saturation | 0.145 | 0.175 | 1 | 0.194 | 0.069 | 1 | 0.179 | 0.094 | 1 |
| Total IgE | -0.739 | 0.058 | 1 | -0.202 | 0.663 | 1 | -0.151 | 0.746 | 1 |
| House dust mite IgE | 0.165 | 0.724 | 1 | 0.070 | 0.882 | 1 | 0.289 | 0.529 | 1 |
| Cat IgE | -0.288 | 0.532 | 1 | -0.693 | 0.084 | 1 | -0.655 | 0.110 | 1 |
| Cathlecidin | 0.002 | 0.989 | 1 | 0.099 | 0.450 | 1 | 0.124 | 0.346 | 1 |
| Gestation period | -0.099 | 0.317 | 1 | -0.077 | 0.436 | 1 | -0.074 | 0.451 | 1 |
| No of children | -0.013 | 0.893 | 1 | -0.037 | 0.707 | 1 | -0.058 | 0.551 | 1 |
| No of siblings | -0.167 | 0.085 | 1 | -0.122 | 0.207 | 1 | -0.111 | 0.252 | 1 |
