# Supplementary material for: Viral respiratory infections and the oropharyngeal bacterial microbiota in acutely wheezing children
Source: PLoS One. 2019 Oct 17;14(10):e0223990. doi: 10.1371/journal.pone.0223990 (PMC6797130; doi:10.1371/journal.pone.0223990)
Supplement: S7 Table — P values adjusted using Bonferonni correction for multiple testing. (DOCX) [file pone.0223990.s007.docx]

S7 Table. Results of Bray-Curtis adonis permutational ANOVA examining clinical variables from individuals with acute wheeze with 99,999 iterations. P values adjusted using Bonferonni correction for multiple testing.

|  | All Acute Wheeze | | | Under 5 years | | |
| --- | --- | --- | --- | --- | --- | --- |
| Clinical variable | r2 | p | p-adjusted | r2 | p | p-adjusted |
| Bacterial biomass | 0.09 | 0 | 0 | 0.061 | 0 | 0 |
| Asthma exacerbation | 0.008 | 0.486 | 1 | 0.011 | 0.606 | 1 |
| Wheezy episode | 0.011 | 0.283 | 1 | 0.019 | 0.188 | 1 |
| viral wheeze | 0.01 | 0.324 | 1 | 0.027 | 0.059 | 1 |
| Bronchiolitis | 0.049 | 0 | 0 | 0.055 | 0.001 | 0.059 |
| URTI | 0.042 | 0 | 0 | 0.046 | 0.004 | 0.236 |
| Age | 0.007 | 0.644 | 1 | 0.012 | 0.579 | 1 |
| Season | 0.021 | 0.032 | 1 | 0.062 | 0.001 | 0.059 |
| Systemic steroids | 0.022 | 0.027 | 1 | 0.028 | 0.059 | 1 |
| Ethnic group | 0.008 | 0.544 | 1 | 0.011 | 0.591 | 1 |
| Platelets | 0.008 | 0.693 | 1 | 0.006 | 0.99 | 1 |
| T-cell count | 0.019 | 0.128 | 1 | 0.016 | 0.597 | 1 |
| Neutraphils | 0.031 | 0.019 | 1 | 0.028 | 0.182 | 1 |
| Lymphocytes | 0.024 | 0.055 | 1 | 0.03 | 0.139 | 1 |
| Monocytes | 0.036 | 0.009 | 0.531 | 0.044 | 0.034 | 1 |
| Eosinophils | 0.023 | 0.063 | 1 | 0.026 | 0.217 | 1 |
| Basophils | 0.005 | 0.943 | 1 | 0.006 | 0.99 | 1 |
| Oxygen required | 0.038 | 0.002 | 0.118 | 0.059 | 0.001 | 0.059 |
| Severity Z-score | 0.016 | 0.174 | 1 | 0.016 | 0.489 | 1 |
| O2saturation | 0.012 | 0.356 | 1 | 0.036 | 0.026 | 1 |
| RV | 0.004 | 0.953 | 1 | 0.011 | 0.584 | 1 |
| RV strain | 0.005 | 0.915 | 1 | 0.009 | 0.799 | 1 |
| RSV | 0.017 | 0.23 | 1 | 0.016 | 0.552 | 1 |
| Adenovirus | 0.024 | 0.08 | 1 | 0.043 | 0.03 | 1 |
| Mycoplasma | 0.024 | 0.211 | 1 | 0.052 | 0.079 | 1 |
| Bordatella | 0.037 | 0.031 | 1 | 0.067 | 0.005 | 0.295 |
| Corona Virus | 0.016 | 0.825 | 1 | 0.027 | 0.718 | 1 |
| hMPV | 0.014 | 0.369 | 1 | 0.016 | 0.605 | 1 |
| Enterovirus | 0.018 | 0.83 | 1 | 0.039 | 0.563 | 1 |
| Bocavirus | 0.353 | 0.1 | 1 | 0.362 | 0.1 | 1 |
| No positive pathogens | 0.007 | 0.58 | 1 | 0.014 | 0.438 | 1 |
| Pathogens tested | 0.007 | 0.614 | 1 | 0.009 | 0.776 | 1 |
| Pathogen positive | 0.009 | 0.411 | 1 | 0.018 | 0.234 | 1 |
| No viruses tested | 0.009 | 0.401 | 1 | 0.012 | 0.528 | 1 |
| No virus positive | 0.006 | 0.7 | 1 | 0.018 | 0.233 | 1 |
| Positive for virus not RV | 0.014 | 0.344 | 1 | 0.01 | 0.878 | 1 |
| No positive viruses, not RV | 0.009 | 0.717 | 1 | 0.017 | 0.536 | 1 |
| No positive viruses | 0.007 | 0.594 | 1 | 0.013 | 0.491 | 1 |
| Atopy | 0.018 | 0.11 | 1 | 0.021 | 0.306 | 1 |
| Total IgE | 0.086 | 0.838 | 1 | 0.083 | 0.839 | 1 |
| House dust mite IgE | 0.164 | 0.423 | 1 | 0.167 | 0.421 | 1 |
| Cat IgE | 0.15 | 0.525 | 1 | 0.156 | 0.531 | 1 |
| Antibiotics | 0.029 | 0.012 | 0.708 | 0.032 | 0.047 | 1 |
| Cathlecidin | 0.018 | 0.332 | 1 | 0.029 | 0.297 | 1 |
| Currently smoking | 0.019 | 0.044 | 1 | 0.027 | 0.064 | 1 |
| Smoking when pregnant | 0.019 | 0.055 | 1 | 0.029 | 0.046 | 1 |
| Smoking regularly when pregnant | 0.007 | 0.658 | 1 | 0.012 | 0.549 | 1 |
| Household smoking | 0.009 | 0.369 | 1 | 0.007 | 0.933 | 1 |
| Gestation period | 0.013 | 0.192 | 1 | 0.022 | 0.131 | 1 |
| No children | 0.007 | 0.695 | 1 | 0.006 | 0.953 | 1 |
| No siblings | 0.012 | 0.214 | 1 | 0.022 | 0.143 | 1 |
| Kindergarten | 0.052 | 0 | 0 | 0.053 | 0.001 | 0.059 |
| Preschool | 0.033 | 0.003 | 0.177 | 0.033 | 0.026 | 1 |
| Daycare | 0.009 | 0.437 | 1 | 0.018 | 0.231 | 1 |
| Recurrence | 0.047 | 0.422 | 1 | 0.064 | 0.656 | 1 |
| Ethnic group | 0.008 | 0.544 | 1 | 0.011 | 0.589 | 1 |
