# Supplementary figures and images for: Viral respiratory infections and the oropharyngeal bacterial microbiota in acutely wheezing children
Source: PLoS One. 2019 Oct 17;14(10):e0223990. doi: 10.1371/journal.pone.0223990 (PMC6797130; doi:10.1371/journal.pone.0223990)

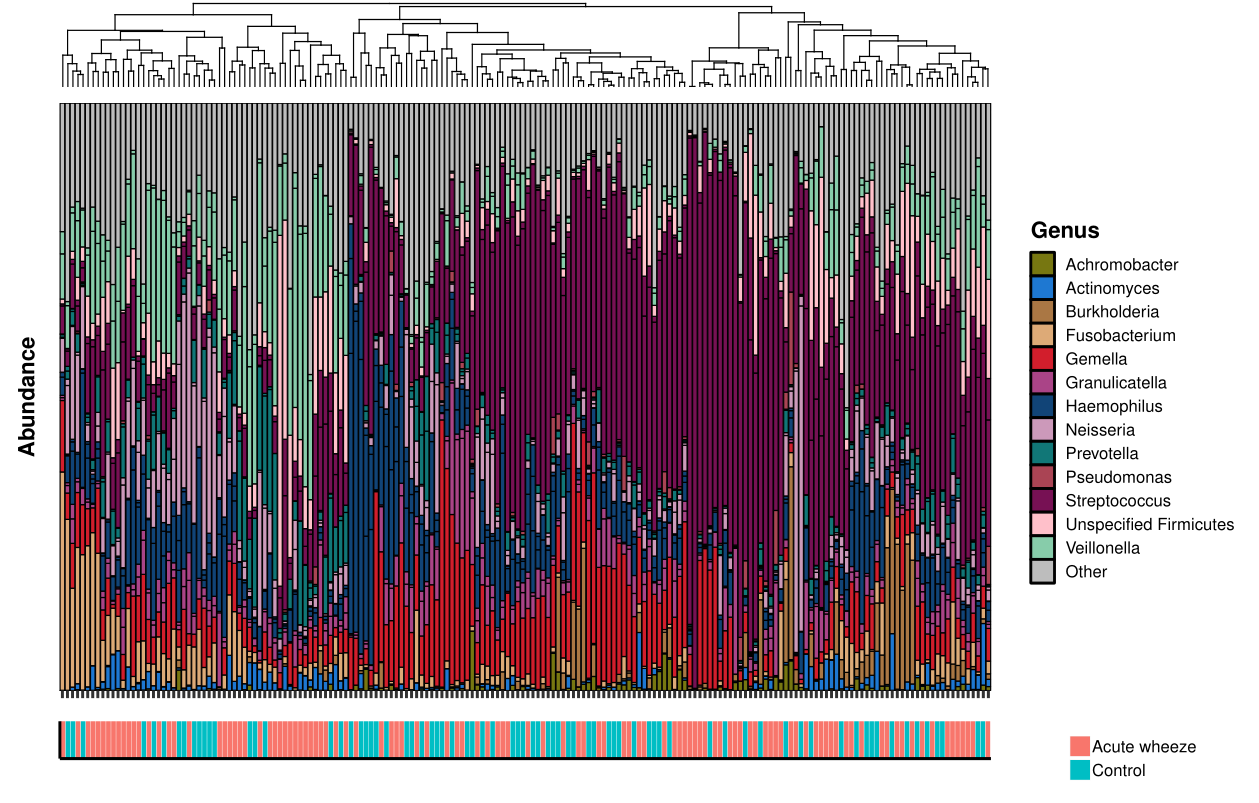

Supplement: S1 Fig — Hierarchical clustering based on Bray-Curtis dissimilarity was used to order stacked bar plots for individuals. Adonis permutational ANOVA explained 1.6% of the variation. (TIFF) [file pone.0223990.s008.tiff]

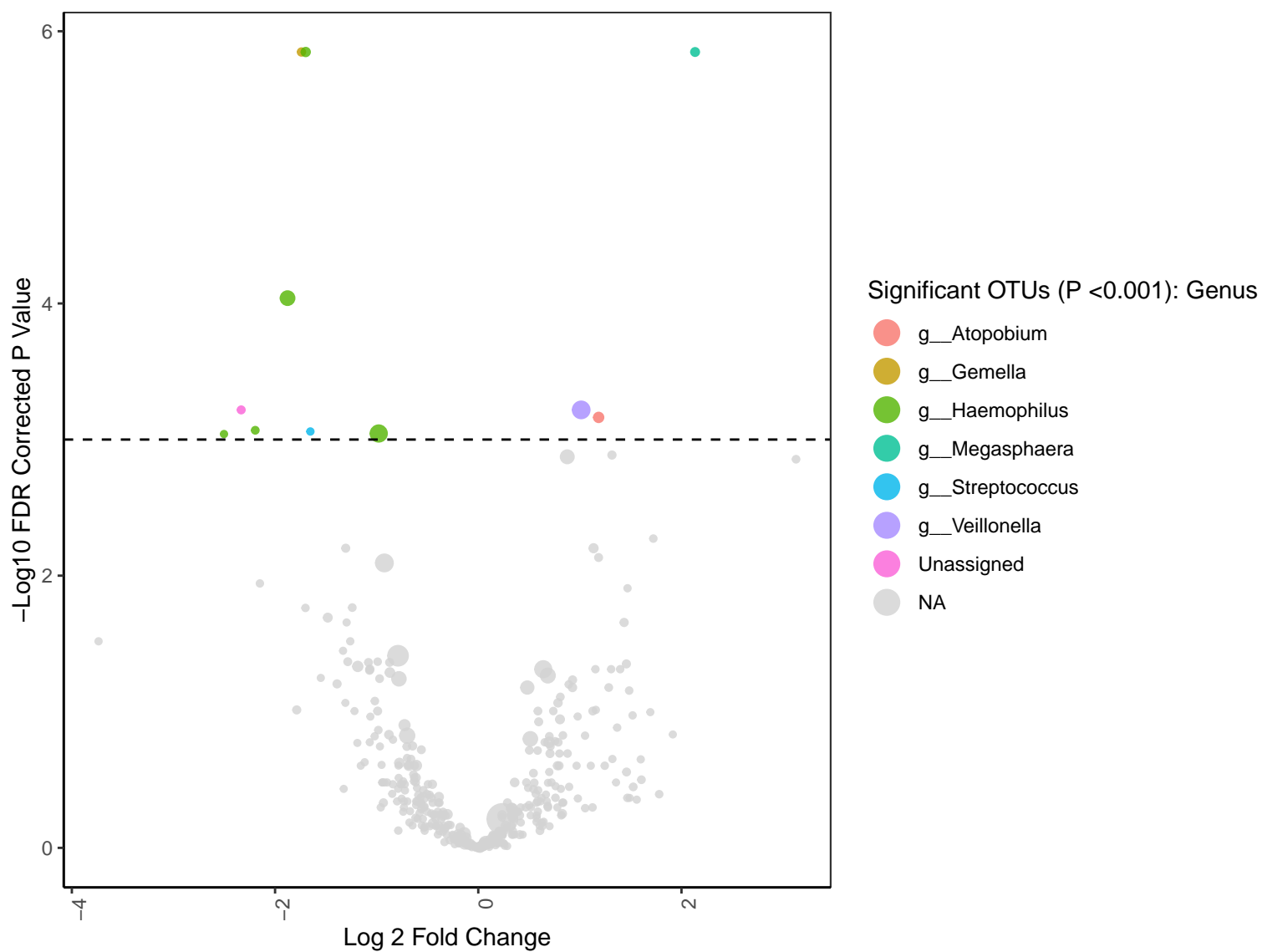

Supplement: S2 Fig — OTUs considered statistically significant (p-value < 0.001) were coloured based on genus level identification. (PDF) [file pone.0223990.s009.pdf]

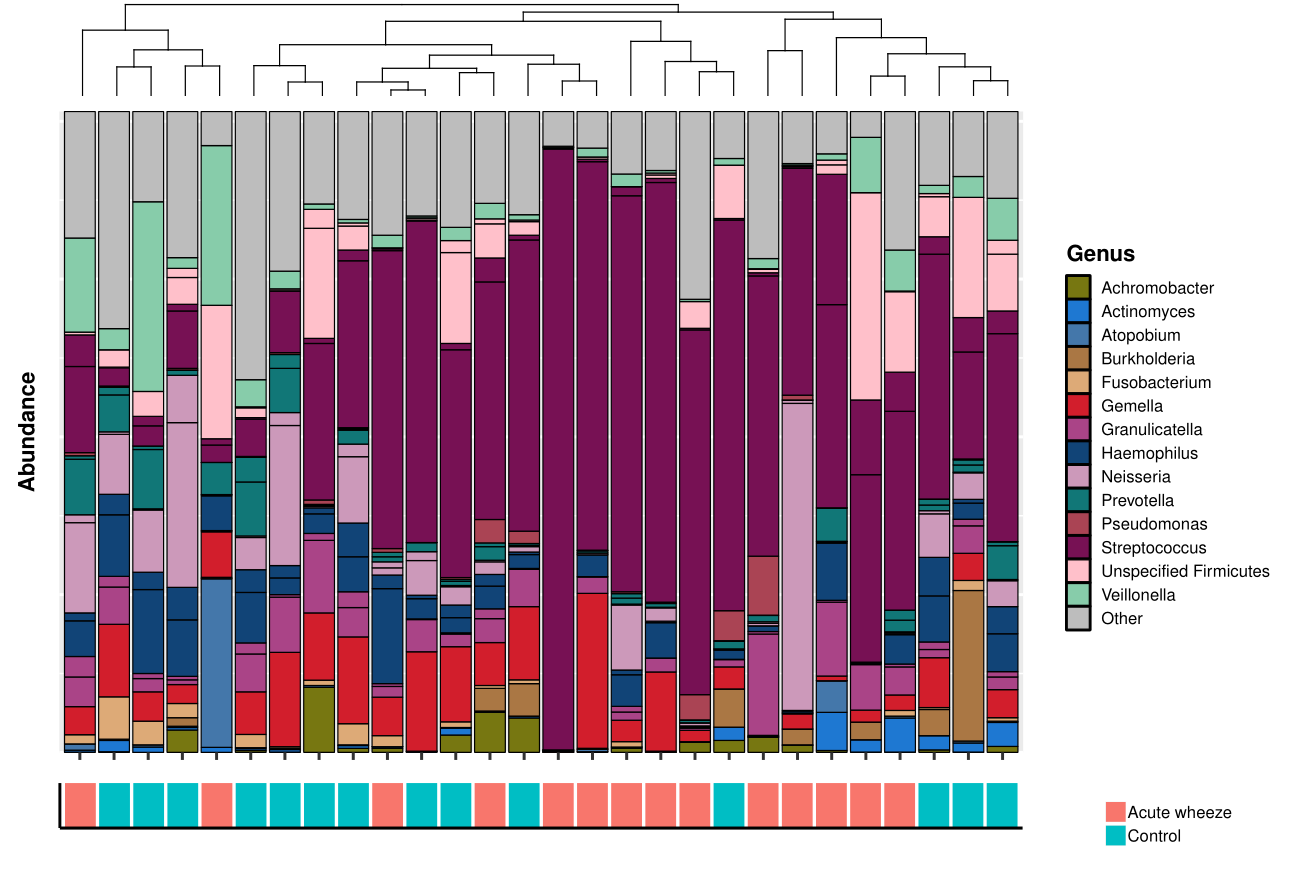

Supplement: S3 Fig — Hierarchical clustering based on Bray-Curtis dissimilarity was used to order stacked bar plots for individuals. Adonis permutational ANOVA revealed bronchiolitis explained 9.8% of the variation. (TIFF) [file pone.0223990.s010.tiff]

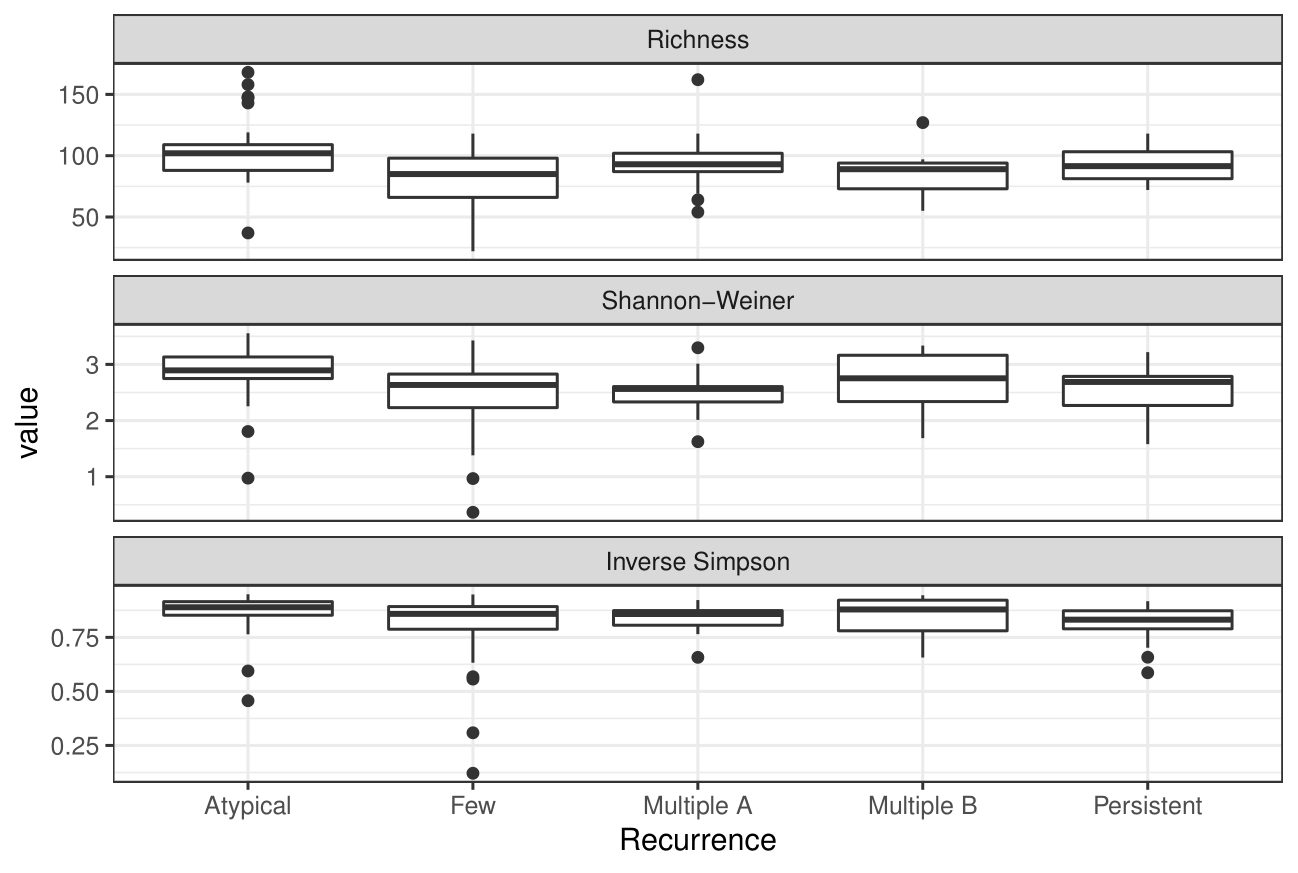

Supplement: S4 Fig — (TIFF) [file pone.0223990.s011.tiff]
