# Supplementary material for: Viral respiratory infections and the oropharyngeal bacterial microbiota in acutely wheezing children
Source: PLoS One. 2019 Oct 17;14(10):e0223990. doi: 10.1371/journal.pone.0223990 (PMC6797130; doi:10.1371/journal.pone.0223990)
Supplement: S1 File — (DOCX) [file pone.0223990.s012.docx]

**Supporting information**

**Viral respiratory infections and the oropharyngeal bacterial microbiota in acutely wheezing children**

Leah Cuthbertson(PhD)^1*^, Stephen WC Oo (MBBS)^2,3*^, Michael J Cox (PhD)^1^, Siew-Kim Khoo (BSc (Hons)^2,4^, Des W Cox (MD)^2^, Glenys Chidlow (PhD)^5^, Kimberley Franks^2,4^, Franciska Prastanti^2,4^, Meredith L Borland (MD)^2,^*^6,7^*, James E Gern (MD)^8^, David W Smith (MBBS)^2,5,9^, Joelene A Bizzintino (PhD)*^2,4^,*  Ingrid A Laing (PhD)*^2,4^*, Peter N Le Souëf (MD*^2,4^*^δ^, Miriam F Moffatt (PhD)^1δ^, William OC Cookson (MD)^1,10δ^

**Supplementary methods**

**Oropharyngeal Swab protocol**

The oropharyngeal throat swab were taken using a sterile plastic shaft swab with a dry cotton or rayon tip. No buffers or gels were present in the swab tube. Swabs were removed from the sample tube immediately before use. Care was taken not to touch surfaces in or out the oral cavity other than the oropharynx. If swab touched any unwanted surface, another swab was used. The middle of the posterior oropharynx was sampled by rubbing the swab 3-5 times around, applying an even pressure and rotating. A tongue depressor may have been used to assist access to the oropharynx.

The swab was immediately placed back into the collection tube and close the lid tightly. Tubes were labelled and stored at -80°C until processing.

**Recurrence**

5 patterns of wheezing observed in this cohort observed included; persistent, multiple A, multiple B, few and atypical.

The *persistent* group was defined as having >6 events over the observation period with >1 acute asthma diagnoses above 5.5 years of age. Usually with multiple events in the last 3.5 years. *Multiple A* was defined as 4-6 events over the observation period, <1 event in last 3.5 years and no acute asthma diagnoses above 5.5 years of age. *Multiple B* was defined as >6 events over the observation period, <1 event in last 3.5 years and no acute asthma diagnoses above 5.5 years of age). *Few* was defined as <3 events over the observation period, <1 event in last 3.5 years and no acute asthma diagnoses above 5.5 years of age. *Atypical* was defined as all those that could not be grouped as above.
