# Supplementary material for: Viral respiratory infections and the oropharyngeal bacterial microbiota in acutely wheezing children
Source: PLoS One. 2019 Oct 17;14(10):e0223990. doi: 10.1371/journal.pone.0223990 (PMC6797130; doi:10.1371/journal.pone.0223990)
Supplement: Attachment — Submitted filename: Response_to_reviewers.docx [file pone.0223990.s013.docx]

Dear Dr Jin,

Thank you for your response to our manuscript. We greatly appreciate the reviewers taking the time to consider this paper for publication in PlosOne, and their very helpful suggestions. We have taken all of their remarks into consideration as follows.

Reviewer #1

We note that the Reviewer considers this to be an interesting research study that would be improved by additions to the bioinformatic and biostatistical analyses. Our responses to specific points are:

*1. QIIME 1 has been succeeded by QIIME 2 because of outdated workflow of QC control and OTU clustering in QIIME 1. The authors should update their analyses by using QIIME 2 or other pipelines that is more advanced than QIIME 1.9.0. This would ideally use amplicon sequence variants instead of 97% OTUs (see this paper for a discussion of this topic:* [*https://www.ncbi.nlm.nih.gov/pubmed/28731476*](https://www.ncbi.nlm.nih.gov/pubmed/28731476)*). Meantime, key parameters of workflow for OTU clustering and taxa assignment would be clearly indicated.*

We are aware of the update of QIIME 1.9.0 to QIIME 2, however QIIME 2 does yet not support dual barcoded data. A work around for this is to use internal illumina demultiplexing, but this process involves the removal of reads pre-analysis and is not optimal. We therefore clarified this reason on line 163 in the methods.

The Reviewer is quite right to draw attention to the potential use of amplicon sequence variants (ASVs). However, ASVs have not been universally accepted, and despite arguments for the use of exact sequence variants (ESVs), OTUs remain an appropriate method of analysis for 16S data. The most important reasons for using OTUs over ASVs or ESVs is that they may lead to the over splitting of OTUs and false inflation of diversity. Considering intragenomic heterogeneity it is possible that multiple ESVs may not be coming from distinct taxa.

We therefore acknowledge this debate in line 177 in the discussion by stating “Exact sequence variants (ESVs) may in some circumstances improve identification of microbial taxa, but genomic sequencing of the airway microbiota is at an early stage. In order to avoid over-splitting of taxa and false inflation of diversity, we have taken the conservative approach of using OTUs in our analyses rather than amplicon sequence variants”.

*2. The authors used 16S V4 region primers and quantitative PCR to measure oral bacterial biomass. If so, was there baseline control for normalization?*

The protocols for performing quantitative PCR (2) have been updated with edits to both the methods and supplementary methods. These updates clarify the procedure used performing oropharyngeal swabs and explain the internal controls implemented during qPCR analysis. Full details of the parameters used for sequencing analysis have been updated in the methods section of the paper on page7, line 148, referencing all parameters used in the analysis.

*3. The authors used three key measures, including Richness, Shannon-Weiner and Inverse Simpsons, to compare the differences of oral microbiota communities between samples from children with and without acute wheeze. However, more detailed comparison of specific bacteria taxa should be performed to show the differential bacterial composition and abundance between cases and controls. For example, as shown in Figure 1, which bacteria(s) was/were the mostly changed in relative abundance; it could be estimated with LEfSe, Wilcoxon signed rank test or Turkey HSD etc*.

We reported the use of Random Forest modelling in the paper to predict OTUs significantly associated with disease states. These models had low predictive power and the model results were not considered reliable (i.e. there is no great difference between the sample groups). Results of indicator species analysis (line 237) and DeSeq2 differential abundance analysis (line 238) have now been presented alongside these results in in line with the Reviewer’s comments.

Discriminant analysis has many assumptions and restrictions, including equal sample sizes and homogeneity of variance/covariance that do not apply here.

*4. The age should be one of important factors influencing oral microbial community. Given the wide range of years of children age (0.08-18.5 yeas), the authors should consider to divide the samples into different ago groups and compare the difference of bacterial composition and abundance.*

We agree with reviewer 1, age is an important factor influencing the microbial community in this study. Several analyses were carried out to investigate the effect of age on the microbial community including controlling for age in the investigation of all clinical characteristics. The cohort of children below the age of 5 were investigated, only bacterial biomass was found to be significantly associated with age in this group. Further groups could not be analysed meaningfully due to the uneven distribution of the age data across the study.

*5. Table 1 lacks statistical comparison of demographic information between cases and controls.*

Statistical comparisons in the form of p-values have been added to Table 1 .

*6. Figure 1 lacks the basic unit of Biomass. The percentage proportion of abundance should be indicated in the y-axis.*

The units on Figure 1 have been updated to express the units of bacterial biomass.

*7. Figure 2 - is it possible that the authors try other PCoA method to cluster samples, for example, weighted or unweighted UniFrac methods?*

We thank reviewer one for suggesting alternative methods of clustering samples based on figure 2. Unweighted UniFrac relies on presences /absence data and does not take into account community composition which can be biased toward rare OTUs. On the other hand, weighted UniFrac uses phylogenetic information to infer genetic relationships based on evolutionary assumptions and this may lead to the introduction of errors.

We have created alternative plots using these measures, but our conclusion is that Bray-Curtis dissimilarity is very commonly used for good reasons, and it remains the most easily understood measure for this plot.

*8. The authors mentioned that "Specific care was made not to contaminate samples with any other part of the mouth". Was there more detailed information?*

The protocols for performing oropharyngeal swabs have been updated with edits to both the methods and supplementary methods (page 7, line 135).

Reviewer #2:

*The article is very relevant and evaluated viral respiratory infections and the oropharyngeal bacterial microbiota in acutely wheezing children. Although they concluded that the microbiota is not associated with viral infection and wheeze in children the article discuss important points that deserve to be published.*

We note with gratitude that the Reviewer considers this to be an interesting research study that would be improved by additions to the bioinformatic and biostatistical analyses. Our responses to specific points are:

*1. It not clear if the children with bronchiolitis are infected with RSV or other virus. This must be explored in the results and in the conclusion. Other important point is to performed and analysis of family and order of the microbiota comparing children with bronchiolitis and the controls.*

We have clarified the viral results associated with bronchiolitis diagnosis at the time of sampling in the results (line 274) and with reference to figure 1 in the manuscript.

*2. Another point that should be explored in the manuscript is breastfeeding and Caesarean delivery, that might be influencing the microbiota.*

We now state on page paragraph that “We did not find any differences in the microbiota that were attributable to recorded breastfeeding. No data was available to explore the effect of delivery method in this study.” Page 13, line 251.

Overall, we feel that the paper has been greatly improved by the Reviewers’ suggestions, and we trust these changes meet with your approval.

Regards,

Dr Leah Cuthbertson
